# The Effectiveness of the RACE Against Suicide Gatekeeper Training on the Knowledge, Perceived Skills, and Attitudes in Suicide Prevention of School Personnel in Higher Education Institutions in the Philippines

**DOI:** 10.1192/j.eurpsy.2026.10690

**Published:** 2026-07-31

**Authors:** I. M. A. F. Divinagracia, K. R. P. Javate

**Affiliations:** Psychiatry, The Medical City, Pasig City, Philippines

## Abstract

**Introduction:**

Suicide remains a major public health challenge among Filipino youth, with rising rates of suicidal ideation and attempts. Schools are critical venues for prevention, yet few locally evaluated gatekeeper training (GKT) programs exist. The RACE Against Suicide GKT was designed to empower school personnel to identify, respond to, and refer at-risk students. While its effectiveness has been demonstrated in basic and secondary education, evidence from higher education institutions (HEIs) is limited.

**Objectives:**

This study evaluated the effectiveness of the RACE Against Suicide GKT program in improving knowledge, perceived skills, and attitudes of HEI personnel toward suicide prevention. It also examined whether outcomes varied by geographic location (Luzon, Visayas, Mindanao), institution type (public, private), and professional role (guidance counselors, non-counselors).

**Methods:**

A quasi-experimental pre-test/post-test design was used among 97 HEI personnel who completed a two-day training. The intervention consisted of three modules on basic suicide prevention, advanced suicide prevention, and postvention strategies delivered through lectures and demonstrations. Outcomes were assessed using true-or-false items for knowledge and self-rated scales for perceived skills and attitudes. Paired t-tests assessed pre- to post-training changes, while independent t-tests and ANOVA examined subgroup differences.

**Results:**

Significant improvements were observed across all domains. The largest gains were in perceived advanced skills (+19.43%, *p*<0.001), basic knowledge (+18.35%, *p*<0.001), and perceived basic skills (+17.22%, *p*<0.001). Advanced knowledge (+9.07%, *p*<0.001) and attitudes (+5.91%, *p*<0.001) also improved. Outcomes did not differ significantly by professional role or institution type, though attitude gains were greater in public institutions. Geographic variation was noted: participants from Visayas showed the largest increase in basic skills but a decline in advanced knowledge, whereas those from Luzon and Mindanao showed consistent improvements.

**Conclusions:**

The RACE Against Suicide GKT effectively enhanced suicide prevention knowledge, skills, and attitudes among HEI personnel. Findings suggest the program is broadly applicable across roles and institutions, though regional tailoring may optimize outcomes. Limitations include self-report measures with variable reliability, self-selection bias, and absence of long-term follow-up. Nevertheless, results support further integration of culturally relevant gatekeeper training into higher education settings and expansion across the school system.

**Disclosure of Interest:**

I. M. A. Divinagracia Paid Instructor of: Unilab Foundation for training activities, K. R. Javate Consultant of: Unilab Foundation

